# Pinpointing Morphology and Projection of Excitatory Neurons in Mouse Visual Cortex

**DOI:** 10.3389/fnins.2019.00912

**Published:** 2019-08-29

**Authors:** Yalun Zhang, Siqi Jiang, Zhengchao Xu, Hui Gong, Anan Li, Qingming Luo, Miao Ren, Xiangning Li, Hao Wu, Jing Yuan, Shangbin Chen

**Affiliations:** ^1^Britton Chance Center for Biomedical Photonics, Wuhan National Laboratory for Optoelectronics-Huazhong University of Science and Technology, Wuhan, China; ^2^MoE Key Laboratory for Biomedical Photonics, School of Engineering Sciences, Huazhong University of Science and Technology, Wuhan, China

**Keywords:** visual cortex, excitatory neuron, morphology, projection pattern, neuron type

## Abstract

The excitatory neurons in the visual cortex are of great significance for us in understanding brain functions. However, the diverse neuron types and their morphological properties have not been fully deciphered. In this paper, we applied the brain-wide positioning system (BPS) to image the entire brain of two Thy1-eYFP H-line male mice at 0.2 μm × 0.2 μm × 1 μm voxel resolution. A total of 103 neurons were reconstructed in layers 5 and 6 of the visual cortex with single-axon-level resolution. Based on the complete topology of neurons and the inherent positioning function of the imaging method, we classified the observed neurons into six types according to their apical dendrites and somata location: star pyramidal cells in layer 5 (L5-sp), slender-tufted pyramidal cells in layer 5 (L5-st), tufted pyramidal cells in layer 5 (L5-tt), spiny stellate-like cells in layer 6 (L6-ss), star pyramidal cells in layer 6 (L6-sp), and slender-tufted pyramidal cells in layer 6 (L6-st). By examining the axonal projection patterns of individual neurons, they can be categorized into three modes: ipsilateral circuit connection neurons, callosal projection neurons and corticofugal projection neurons. Correlating the two types of classifications, we have found that there are at least two projection modes comprised in the former defined neuron types except for L5-tt. On the other hand, each projection mode may consist of four dendritic types defined in this study. The axon projection mode only partially correlates with the apical dendrite feature. This work has demonstrated a paradigm for resolving the visual cortex through single-neuron-level quantification and has shown potential to be extended to reveal the connectome of other defined sensory and motor systems.

## Introduction

The visual system of mammals receives most of the sensory information. The understanding of visual circuits requires the morphologic characterization and classification of individual neurons (Liang et al., 2015). Recently, tremendous progress has been achieved in studying the projection mode of the mouse visual cortex (Kim et al., 2015; Kondo and Ohki, 2016; Roth et al., 2016; Sun et al., 2016; Atlan et al., 2017). A mouse mesoscale connectome among different brain regions has been constructed by combining adeno-associated virus (AAV) tracer labeling and serial two-photon (STP) imaging (Oh et al., 2014). With both rabies virus and AAV co-injection, it is possible to study the input and output connection of the visual neural network (Zhang et al., 2016). However, most studies perform only bulk tracing of gross projection patterns for a large number of neuronal populations (Jeong et al., 2016). The revealed region-level connectivity fails to reflect the collateral arborization patterns of each projection neuron (Mitra, 2014). Our previous work has demonstrated the feasibility of performing single-axon-level morphological analysis of projection neurons in the mouse barrel cortex and secondary motor cortex (Guo C. et al., 2017; Lin et al., 2018; Sun et al., 2019). Thus, it is possible to explore the excitatory neurons of the visual cortex at single-neuron resolution.

Advances in techniques have boosted the morphological analysis of individual neurons (Molnar and Cheung, 2006; Ascoli et al., 2008; Costa et al., 2010; Meyer et al., 2010; Oberlaender et al., 2012; Huang, 2014). Molyneaux et al. (2007) reviewed the major subtypes of projection neurons classified by axonal hodology. Fame et al. (2011) summarized the specification and diversity of callosal projection neurons. Oberlaender et al. (2012) defined excitatory neuron types in the rat barrel cortex based on morphological features. Markram et al. (2015) reconstructed masses of neurons with the cortical part morphology and classified them according to their location of somata and morphology. Nevertheless, the long-range projection neurons are not sufficiently available for linking dendritic classification and axonal hodology (Gouwens et al., 2018; Kanari et al., 2019). To pinpoint the excitatory neurons in the visual cortex, it is timely to study their complete morphology, neuron types and corresponding projection patterns.

Here, we applied the brain-wide positioning system (BPS) (Gong et al., 2016) to obtain volumetric imaging of the whole mouse brain dataset with a resolution of 0.2 μm × 0.2 μm × 1 μm. A total of 103 excitatory neurons in layers 5 and 6 were reconstructed. Due to the inherent positioning feature of the BPS system, it is possible to localize each neuron’s soma and its neurites at the nucleus level. This will allow us to quantify the morphology, neuron types and projection patterns with unprecedented precision.

## Materials and Methods

### Tissue Preparation

All the animal experiments followed procedures that had been approved by the Institutional Animal Ethics Committee of Huazhong University of Science and Technology. Animal care and use were performed in accordance with the guidelines of the Administration Committee of Affairs Concerning Experimental Animals in Hubei Province of China. Two 8-week-old Thy1-eYFP H-line transgenic male mice (Jackson Laboratory, Bar Harbor, ME, United States) were used (Feng et al., 2000; Porrero et al., 2010). All histological procedures had been previously described (Gang et al., 2017; Guo W. et al., 2017). Briefly, the mouse was anesthetized with a 1% solution of sodium pentobarbital and subsequently intracardially perfused with 0.01 M PBS (Sigma-Aldrich Inc., St. Louis, MO, United States), followed by 4% paraformaldehyde (Sigma-Aldrich Inc., St. Louis, MO, United States). The whole brain was excised and post-fixed in 4% paraformaldehyde at 4°C for 24 h. Then, the brain was rinsed overnight at 4°C in 0.01 M PBS and subsequently dehydrated in a graded ethanol series (50, 70, and 95% ethanol at 4°C for 1 h each). After dehydration, the brain was immersed in a series of graded glycol methacrylate (GMA) (Ted Pella Inc., Redding, CA, United States) including 0.2% Sudan Black B (SBB): 70, 85, and 100% GMA for 2 h each and 100% GMA overnight at 4°C. The brain sample was impregnated in a prepolymerized solution of GMA for 3 days at 4°C and finally embedded in a vacuum oven at 48°C for 24 h. The 100% GMA solution contained 67 g A solution, 29.4 g B solution, 2.8 g deionized water, 0.2 g SBB, and 0.6 g AIBN as initiator. The 70 and 85% GMA (wt/wt) were prepared from 95% ethanol and 100% GMA.

### Whole-Brain Imaging

The resin-embedded whole-brain sample that could provide a certain hardness was sectioned and imaged automatically using BPS. Before imaging, we immobilized the mouse whole-brain sample in the anterior-posterior direction in a water bath on a 3D translation stage. The water bath was filled with 0.01 M Na_2_CO_3_ and propidium iodide (PI) solution, in which the sample was immersed to provide a matched refractive index for the objective lens during imaging. In addition, the PI molecules could quickly stain nucleic acids inside the cell body to provide the position of the cell, and the Na_2_CO_3_ solution could enhance the fluorescence of eYFP. Sectioning was achieved using a fixed diamond knife and a 3D translation stage for wide-field large-volume tomography. The single sectioning thickness was set to 2 μm, and the sectioning width was 2 mm. The imaging was performed using a 20× water immersion objective on a fast structured illumination microscope (1.0 NA, XLUMPLFLN 20XW, Olympus, Shinjuku, Tokyo, Japan). The imaging plane was set below the surface of the sample block. The eYFP and PI molecules were excited simultaneously, and the emitted fluorescence signals were separated by a dichroic mirror and detected by two cameras. During the imaging process, we performed axial scanning using the piezoelectric translational stage, which acquired two sectioning images at depths of 1 and 2 μm. Following axial scanning, the sample was moved to the next mosaic field of view (FOV), with a 10 μm overlap between adjacent FOVs. The mosaic imaging process was repeated until the entire coronal section was acquired. Finally, we acquired the dataset sections with a 1 μm thickness and performed imaging with a voxel size of 0.2 μm × 0.2 μm × 1 μm.

### Image Processing

Image preprocessing was applied to standardize the datasets. First, the tiles at the same position of the eYFP and PI channels were resized and shifted to obtain the perfectly matched tiles by the parameters of the model experiment. Second, we stitched the tiles of the same section to obtain a mosaic section based on the accurate spatial orientation and neighboring overlap (approximately 10 pixels). The anchor points of the tiles were spaced equally in two orthogonal directions. Third, transverse illumination correction was performed separately in each section. Fourth, axial illumination correction was performed based on the average intensity of each section. The two illumination correction steps were based on our previously developed algorithm (Ding et al., 2013). Finally, we obtained the standard dataset for both the eYFP and PI channels. Image preprocessing was implemented with C++, parallel optimized using the Intel MPI Library and then executed on a computing server (72 cores, 2 GHz/core) for 6 h for each mouse brain dataset at a voxel resolution of 0.2 μm × 0.2 μm × 1 μm.

### Reconstruction and Statistics

**FIGURE 1:**
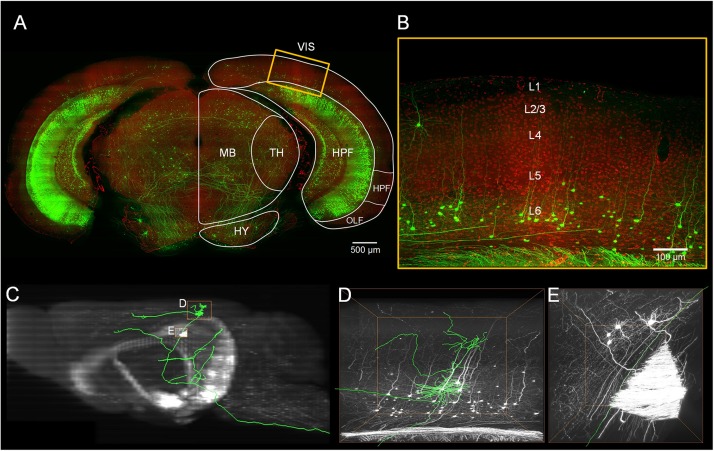
Localization of the visual cortex and annotating the individual neurons inside. **(A)** Merged image with green channel (thickness of projection: 500 μm) and red channel (thickness of projection: 5 μm). The white outline indicates brain regions: the visual areas (VIS), the hippocampal formation (HPF), the thalamus (TH), the midbrain (MB) and the hypothalamus (HY). Scale bar: 500 μm. **(B)** Enlarged view of the region marked with the yellow rectangle in **(A)**. The YFP-labeled individual neurons and the propidium iodide (PI)-stained cytoarchitecture are shown. Scale bar: 100 μm. **(C)** The maximal projection of the sagittal plane of the green channel dataset. **(D,E)** Three-dimensional enlarged view of the region marked with the yellow rectangle in **(C)**.

**FIGURE 2:**
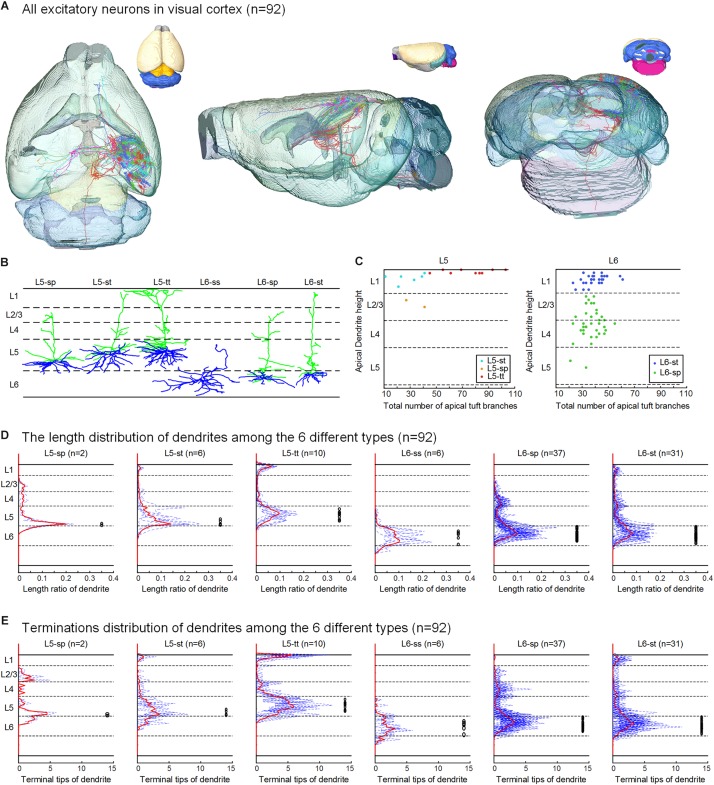
Excitatory neurons in the visual cortex and their classification based on dendritic features. **(A)** The 92 neurons with their complete morphologies are shown in the mouse brain, the six colors of neurons represent the six types defined in **(B)**, and the different colors of the mouse brain represent the mapped brain regions according to the Allen Reference Atlas. **(B)** According to the layer of the cell body and the dendrite morphology, the reconstructed neurons are separated into six categories: star pyramidal cell in layer 5 (L5-sp), slender-tufted pyramidal cell in layer 5 (L5-st), tufted pyramidal cell in layer 5 (L5-tt), spiny stellate-like cell in layer 6 (L6-ss), star pyramidal cell in layer 6 (L6-sp), and slender-tufted pyramidal cell in layer 6 (L6-st). **(C)** Scatter plots with the two parameters: the relative height of the apical dendrites and the number of apical dendrites. The L5-sp, L5-st, L5-tt, L6-sp, and L6-st neurons show relatively clustered groups. **(D)** Laminar percentage distribution of dendritic L5-sp, L5-st, L5-tt, L6-ss, L6-sp, and L6-st neurons. For each type of neuron, the solid red line is the mean result, and the black circles to the right indicate the location of somata. **(E)** Laminar distribution of dendrite terminals of the 6 types of neurons.

We localized the mouse’s visual cortex in the PI channel image series from the Thy1-eYFP H-line datasets by referring to the Allen Mouse Brain Reference Atlas^1^ (Figure 1). We manually reconstructed 103 neurons through man-machine interactive annotation in layers 5 and 6 of the visual cortex from the whole brain fluorescence imaging dataset using the Amira software (FEI, Mérignac Cedex, France) with a homemade TDat plugin (Li et al., 2017). Every neuron was reconstructed and checked back-to-back. The morphological information was saved as a ^∗^.swc file. Meanwhile, we parceled the layers of the cortex on the PI channel image series based on the cytoarchitectural features. We corrected the neurons based on the normal vector of the cortex’s layers and located the position of the neuron’s soma and dendrites by the location of the cortex’s layers. Based on the PI channel image series according to the Allen Reference Atlas, we mapped the brain regions (Figure 2A) and determined the route and terminal location of the axon fibers. All morphology parameters were measured using Neurolucida Explorer (MBF Bioscience, Williston, VT, United States). For the morphological statistics (Tables 1, 2), we performed multiple group comparisons assessed with one-way ANOVA followed by *post hoc* Tukey’s test. These analyses were performed using the SPSS software (v22, IBM, New York, NY, United States).

**TABLE 1:** Morphological statistics of excitatory neurons in layer 5 of visual cortex.

	**L5-sp(*n* = 2)**	**L5-st(*n* = 6)**	**L5-tt(*n* = 10)**
Length of apical dendrite (μm)	1453.2 ± 7.3	1711.8 ± 288.7	3780.3 ± 259.5
Branches of apical dendrite	34.0 ± 7.0	28.0 ± 4.2	73.5 ± 5.3
Max branch order of apical dendrite	12.5 ± 1.5	9.2 ± 1.2	20.1 ± 1.2
Terminal tips of apical dendrite	17.5 ± 3.5	14.5 ± 2.1	37.3 ± 2.8
Length of basal dendrite (μm)	1428.4 ± 683.0	2468.8 ± 225.2	3271.7 ± 224.7
Branches of basal dendrite	28.5 ± 9.5	38.7 ± 3.5	66.6 ± 4.3
Max branch order of basal dendrite	4.0 ± 1.0	5.0 ± 0.4	5.8 ± 0.2
Terminal tips of basal dendrite	17.0 ± 4.0	22.5 ± 1.9	37.7 ± 2.2
Axonal length (μm)	6798.6 ± 682.4	12585.7 ± 2904.0	16694.0 ± 3498.6
Axonal branches	14.0 ± 1.0	104.3 ± 19.9	68.6 ± 17.9
Max branch order of axon	6.0 ± 1.0	20.7 ± 3.0	10.3 ± 1.8
Terminal tips of axon	7.5 ± 0.5	52.7 ± 10.0	34.8 ± 9.0

**TABLE 2:** Morphological statistics of excitatory neurons in layer 6 of visual cortex.

	**L6-ss(*n* = 6)**	**L6-sp(*n* = 37)**	**L6-st(*n* = 31)**
Length of apical dendrite (μm)	–	1595.4 ± 48.1	1898.9 ± 66.5
Branches of apical dendrite	–	36.6 ± 1.3	40.0 ± 1.5
Max branch order of apical dendrite	–	14.6 ± 0.5	16.1 ± 0.5
Terminal tips of apical dendrite	–	18.8 ± 0.6	20.6 ± 0.8
Length of basal dendrite (μm)	2922.1 ± 203.2	1080.4 ± 41.2	1213.7 ± 62.4
Branches of basal dendrite	45.0 ± 3.5	25.7 ± 1.1	26.3 ± 1.4
Max branch order of basal dendrite	7.7 ± 1.0	4.2 ± 0.1	4.0 ± 0.2
Terminal tips of basal dendrite	25.8 ± 2.0	15.9 ± 0.6	16.4 ± 0.7
Axonal length (μm)	5964.2 ± 688.2	6720.7 ± 358.3	8021.1 ± 481.9
Axonal branches	49.7 ± 11.8	40.3 ± 6.9	47.2 ± 5.7
Max branch order of axon	14.5 ± 3.2	10.4 ± 1.2	11.5 ± 1.0
Terminal tips of axon	25.3 ± 5.9	20.6 ± 3.5	24.2 ± 2.9

## Results

### Dendritic Classification of Neuron Types

Using the BPS system (Gong et al., 2016), we obtained two whole brain datasets of Thy1-eYFP H-line male mice with a voxel resolution of 0.2 μm × 0.2 μm × 1 μm (Figure 1 and Supplementary Figure 1). With the aid of PI staining, it is feasible to parcel the brain regions and cortex layers (Figures 1B, 2A). A total of 103 excitatory neurons were randomly selected and reconstructed in layers 5 and 6 (L5 and L6) of the visual cortex (Figure 2A and Supplementary Figure 1). The 92 neurons confirmed in the visual cortex (Supplementary Table 1) of the same mouse brain were analyzed and classified with details. The 11 neurons from another mouse brain were used to validate the classification. The inherent positioning function of BPS allows us to precisely locate the soma and neurite extension in the whole brain space and quantify the reconstructed morphology.

According to the laminar position of the soma and apical dendritic morphology (Molnar and Cheung, 2006; Oberlaender et al., 2012; Guo C. et al., 2017), we divided the 92 neurons into six categories: 2 star pyramidal cells in layer 5 (L5-sp), 6 slender-tufted pyramidal cells in layer 5 (L5-st), 10 tufted pyramidal cells in layer 5 (L5-tt), 6 spiny stellate-like cells in layer 6 (L6-ss), 37 star pyramidal cells in layer 6 (L6-sp), and 31 slender-tufted pyramidal cells in layer 6 (L6-st) (Figure 2B). The dendrites of the st- and tt-type reach the first layer of the cortex, while the tt-type has tufted apical dendrites. The dendrites of sp-type do not reach the first layer. The ss-type has no typical apical dendritic morphology of the pyramidal neurons (Figure 2B). Considering the significant differences in thickness among the wide span of the visual cortex, laminar normalization of layer thickness was performed before quantifying the apical dendrite morphology. The total number of branches and the height of the apical dendrites clearly showed five groups corresponding to the five types of neurons: L5-sp, L5-st, L5-tt, L6-sp, and L6-st (Figure 2C). Including the basal dendrites, the length distribution of the dendrites among the 6 different types was analyzed (Figure 2D). The results showed that (1) L5-sp neurons have a peak dendritic length ratio in L5 and a zero value in L1; (2) L5-st neurons have a peak distribution in L5 and a small ratio in L1; (3) L5-tt neurons show a bimodal distribution of dendritic length in L5 and L1; (4) L6-ss neurons present a wide peak in the L6 layer but without distribution in L4, L23, or L1; (5) L6-sp neurons have an obvious distribution from L4 to L6 but not L1; and (6) L6-st-type neurons show a peak distribution in L6 and a small ridge in L1. The laminar distribution of the dendrite terminals of the six types of neurons (Figure 2E) has a similar tendency to the corresponding length distribution. In addition, the L5-tt neuron presented the most complex arborization and the longest total length of neurites among all the abovementioned types (Tables 1, 2).

Even for the 11 neurons from the visual cortex of the second mouse brain, we found all 6 dendrite-defined types with consistent morphological features: 1 L5-sp, 2 L5-st, 1 L5-tt, 2 L6-ss, 3 L6-sp, and 2 L6-st (Supplementary Figure 1).

### Axonal Classification and Projection Patterns

**FIGURE 3:**
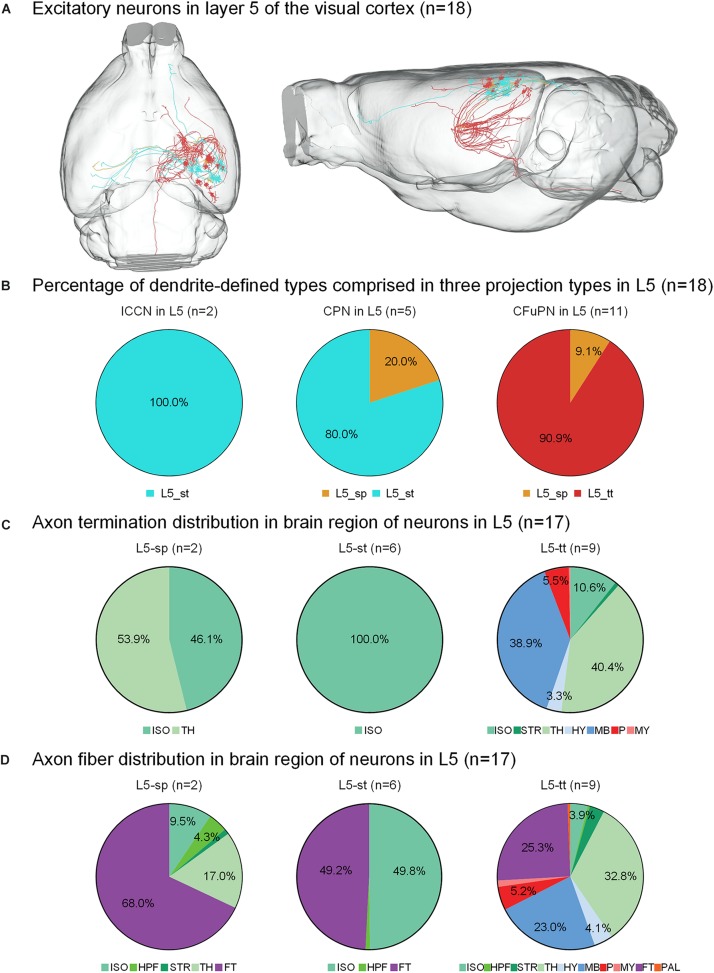
Axonal classification and projection patterns of 18 excitatory neurons in layer 5. **(A)** The 18 neurons in L5 with their complete morphologies are shown in the mouse brain. **(B)** The percentage of dendrite-defined types comprises the ipsilateral circuit connection neurons (ICCNs), the callosal projection neurons (CPNs) and the corticofugal projection neurons (CFuPNs) in L5. **(C)** The pie charts show the regional distribution of axonal terminals for different neuronal types in L5. **(D)** The pie charts show the percentage distribution of axonal length in different brain regions for the L5 neurons. For brain regions: isocortex (ISO), hippocampal formation (HPF), striatum (STR), thalamus (TH), hypothalamus (HY), midbrain (MB), pons (P), medulla (MY), fiber tracts (FT), and pallidum (PAL).

**FIGURE 4:**
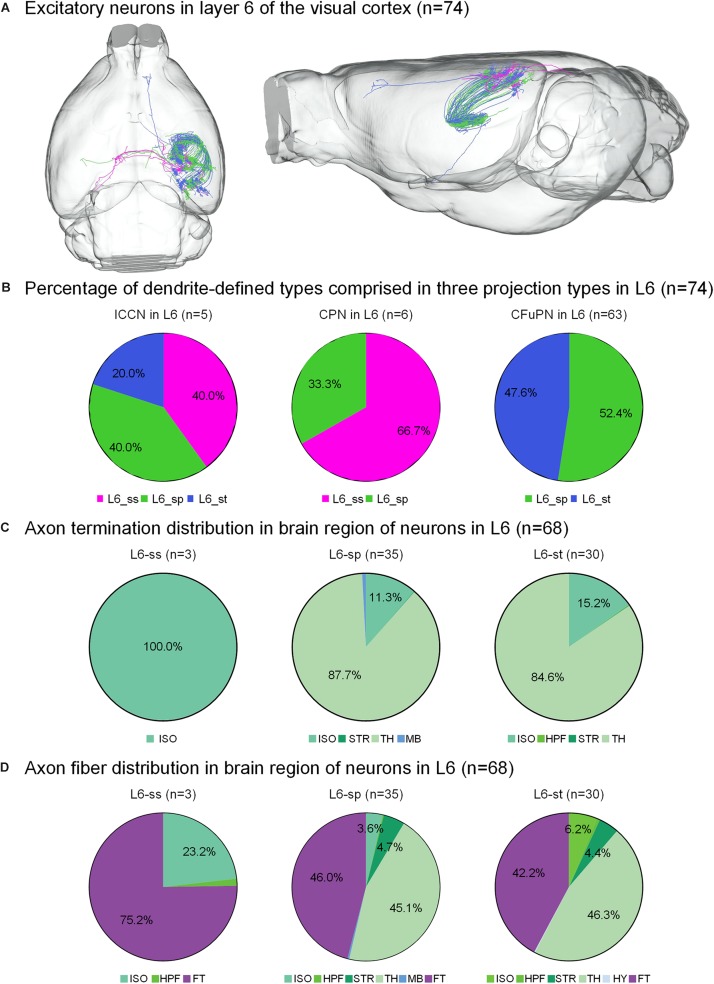
Axonal classification and projection patterns of 74 excitatory neurons in layer 6. **(A)** The 74 neurons in L6 with their complete morphologies are shown in the mouse brain. **(B)** The percentage of dendrite-defined types comprises the ICCNs, CPNs and CFuPNs in L6. **(C)** The pie charts show the regional distribution of axonal terminals for different neuronal types in L6. **(D)** The pie charts show the percentage distribution of axonal length in different brain regions for the three types of L6 neurons.

Because the 92 neurons are long-range excitatory neurons, the projection paths and terminals of their axons were analyzed. In addition to neuronal dendrite-defined types, 92 neurons could be classified as 7 ipsilateral circuit connection neurons, 11 callosal projection neurons and 74 corticofugal projection neurons (CFuPNs) based on axonal extension (Molyneaux et al., 2007; Fame et al., 2011; Greig et al., 2013). The axonal classification and projection patterns were separated into the L5 and L6 groups and are shown in Figures 3, 4. To make the results more rational, we excluded neurons with the ratio of pretended termination in a fiber tract to all the terminations over 0.1 from the terminal distribution analyses (Figures 3C,D, 4C,D). Thus, 17 L5 neurons and 68 L6 neurons were pooled for the quantification.

For the 18 neurons in layer 5 of the visual cortex, the L5-tt type only has corticofugal projections, while the L5-sp and L5-st types of neurons have two possible projection modes (Figure 3B). For example, L5-st neurons match both the typical subtypes of ipsilateral circuit connection neuron and callosal projection neuron (Fame et al., 2011), and the L5-sp is distributed into the callosal projection neuron and CFuPN subtypes. Along with the difference in axon types, their projection patterns are also significantly different (Figure 3C). The projection terminals of L5-st are mainly concentrated in the isocortex, presenting possible intracortical regulation. The L5-sp and L5-tt types of neurons all have subcortical projection targets. L5-sp mainly extends axon terminals in thalamus, while the L5-tt type shows frequent terminals in both thalamus and midbrain for over 30% of the distribution. On the other hand, the percentage of axon length in different brain regions was also calculated for all neurons (Figure 3D). The main distributions of L5-st are in the fiber tracts and isocortex. L5-sp has a large number of axon distributions in the fiber tracts and thalamus, and L5-tt axons are distributed in thalamus, midbrain, and the fiber tracts all at greater than 20%. Specifically, an L5-st neuron spreads its axon to the anterior part of the cortex and projects to the orbital area, lateral part (Figure 3A).

The 74 neurons in layer 6 can be divided into three categories: 6 L6-ss, 37 L6-sp and 31 L6-st. The L6-ss, L6-sp, and L6-st types of neurons all have at least two possible projection modes (Figure 4B). For example, L6-ss neurons match both the typical subtypes of ipsilateral circuit connection neuron and callosal projection neuron. The L6-st match both the ipsilateral circuit connection neuron and CFuPN subtypes. The L6-sp can be distributed into all the ipsilateral circuit connection neuron, callosal projection neuron and CFuPN subtypes. Although the axon types of excitatory neurons in L6 are similar to those of excitatory neurons in L5, they have differences in projection patterns (Figure 4C). The L6-sp and L6-st types of neurons all have subcortical projection targets and mainly extend axon terminals in thalamus for over 80% of the distribution, and the projection terminal of L6-ss is mainly concentrated in the isocortex, presenting possible intracortical regulation the same as L5-st. For the axon fiber distribution in the brain region of 74 neurons in L6, the fiber tracts and thalamus both present a length distribution of more than 40% in the L6-sp and L6-st types (Figure 4D). In particular, the main distributions of L6-ss are in the fiber tracts and the isocortex. An L6-st neuron spreads its axon to the anterior part of the cortex and projects to both the secondary motor area and anterior cingulate area, dorsal part (Figure 4A).

Similarly, the 11 neurons from the second mouse brain can be classified into 3 axonal types: 8 CFuPNs, 2 callosal projection neurons and 1 ipsilateral circuit connection neuron. They also present typical axon projections to different brain regions (Supplementary Figure 1).

### Axon Targets of the Corticofugal Projection Neurons (CFuPNs)

**FIGURE 5:**
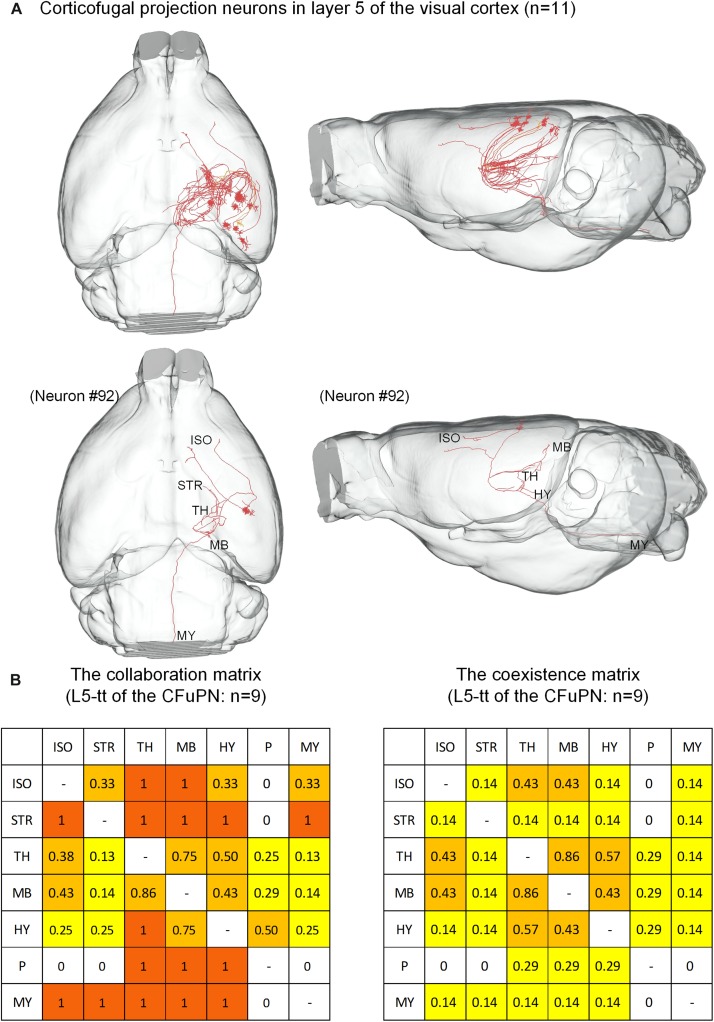
Distribution of axon terminals for the CFuPN in L5. **(A)** In the panel of top row, the 11 CFuPN in L5 with their complete morphologies are shown in the mouse brain. In the panel of bottom row, a typical neuron (#92) presents multiple axonal terminals in different brain regions. **(B)** The L5-tt of CFuPN: *n* = 9. The collaboration matrix of L5-tt of the CFuPN quantifies the “posterior probability” of another projection region given the neuron projection to the current region. Taking the values in the first row and second column as examples, “0.33” means 33% of the neurons projected to isocortex project to striatum. The values in the first row and third column: “1” means the neurons projected to isocortex must project to thalamus. The coexistence matrix of L5-tt of the CFuPN measures the occurrence frequency of two directional projection modes. Every element of the matrix represents the ratio of some neurons projecting to the two regions to all the neurons having at least two projection regions.

The 74 CFuPNs consist of 1 L5-sp, 10 L5-tt, 33 L6-sp and 30 L6-st neurons. Both the CFuPNs in L5 and L6 exhibit multi-region projections. For the 11 CFuPNs in L5, 9 neurons of the 10 L5-tt neurons was analyzed using the collaboration and coexistence matrices defined in our previous work (Guo C. et al., 2017) (Figure 5). We found that thalamus, midbrain and hypothalamus are the most popular targets of the CFuPNs in L5. The axon terminals of individual L5-tt neuron are found in at least two or more brain regions. All the axon terminals of the L5-tt type in this work are distributed in seven brain regions. Here, the axon-defined type of L5-tt should be subcerebral projection neurons introduced in the references (Molyneaux et al., 2007).

**FIGURE 6:**
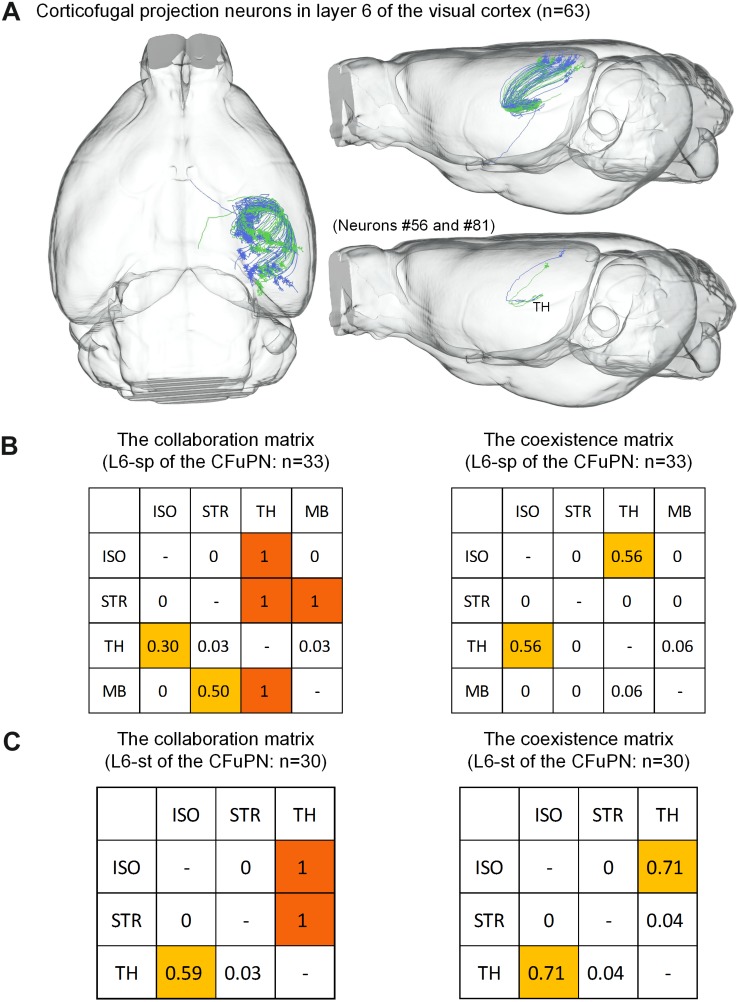
Distribution of axon terminals for the CFuPN in L6. **(A)** The 63 CFuPN in L6 with their complete morphologies are shown in the mouse brain. In the bottom right of panel **(A)**, two representative L6-sp (#56) and L6-st (#81) neurons show their axonal terminals in thalamus. **(B)** The collaboration and coexistence matrices of L6-sp. L6-sp in corticofugal projection neurons: *n* = 33. **(C)** The collaboration and coexistence matrices of L6-st. L6-st in corticofugal projection neurons: *n* = 30.

For the 63 CFuPNs in L6, the L6-sp type consisting of 33 neurons and the L6-st type consisting 30 neurons are quantified using the matrices (Figure 6). We found that thalamus are the most popular targets of the two types of neurons. Almost all the neurons that project to other brain regions have terminal points in thalamus at the same time. According to the features of long-range neurons (Molyneaux et al., 2007; Harris and Shepherd, 2015), the L6-sp and L6-st of the CFuPNs belong to corticothalamic neurons.

### Correlates Between Dendritic Types and Axon Projection Patterns

**FIGURE 7:**
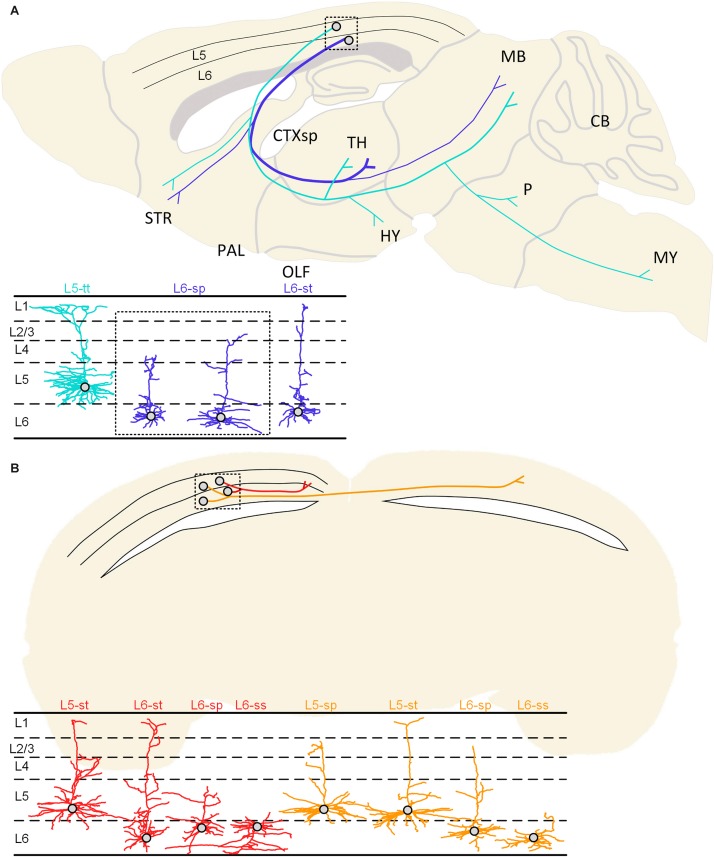
Summarized projection patterns of the studied neurons in the visual cortex. **(A)** Two typical projection patterns of corticofugal projection neurons. The cyan-colored neuron shows a cell body located in L5 of the cortex and the subcerebral projection mode. The same cyan-colored dendrites in the lower left corner indicate the L5-tt type. The blue colored neuron presents soma in the L6 and the corticothalamic projection mode. The blue-colored dendrites may be classified as L6-sp and L6-st types according to the dendritic morphology. **(B)** The projection patterns of the ipsilateral circuit connection neurons and the callosal projection neurons. The ipsilateral circuit connection neurons (red) with somata located in the 5th or 6th layer of the cortex can be classified as L5-st, L6-st, L6-sp, and L6-ss dendritic types. The callosal projection neurons (orange) with cell bodies located in layer 5 or 6 can be distinguished as L5-sp, L5-st, L6-sp, and L6-ss types.

Taking the aforementioned results together, we could form a wiring map with cell-type specificity. For the CFuPN subtype, we found that they had two typical projection patterns. The L5-tt neurons have the subcerebral projection mode only, and they spread their axons to several brain regions, for example, striatum, thalamus, hypothalamus, midbrain, pons and medulla. The corticofugal projection neurons of the L6-st and L6-sp have the corticothalamic projection mode. The corticofugal projection neurons of the L6-st and L6-sp types could distribute their axons to striatum, thalamus, and midbrain, and most of them project to thalamus (Figure 7A).

The ipsilateral circuit connection neuron and callosal projection neuron located in L5 and L6 have undistinguishable dendrite features but have different axon projection patterns. The axons of the ipsilateral circuit connection neurons are distributed in the ipsilateral hemisphere and do not cross the corpus callosum. These neurons with somata located in the 5th or 6th layer of the cortex can be classified as L5-st, L6-st, L6-sp, and L6-ss dendritic types. The axons of the callosal projection neurons are distributed across the corpus callosum to the contralateral hemisphere, and their fibers and terminals are distributed in both the ipsilateral and contralateral hemisphere. The callosal projection neurons with cell bodies located in layer 5 or 6 can be distinguished as L5-sp, L5-st, L6-sp, and L6-ss types (Figure 7B).

Considering the classified neuron types and the corresponding distribution of axon projections, it is easy to notice a partial correlation between the neuron dendrite complexity and the axon projection mode.

## Discussion

Digital reconstruction of neuron morphology is non-trivial in neuroscience (Parekh and Ascoli, 2013). Considering the bottleneck presented by the low speed of construction, the total number of 103 neurons from the visual cortex is relatively large. This is a systematic work on neuroanatomy with single-neuron resolution. Based on the BPS (Gong et al., 2016), we could achieve not only high resolution (voxel resolution of 0.2 μm × 0.2 μm × 1 μm) but also coherent positioning functions. This work demonstrates an alternative platform for single-neuron mapping, reconstruction and quantification (Albanese and Chung, 2016; Economon et al., 2016; Gong et al., 2016). In this way, we could pinpoint the dendrite features and axon projection patterns without ambiguity, and correlate the dendritic classification and axonal hodology. This is one of a series of works by the authors on single-neuron mapping (Gong et al., 2016; Guo C. et al., 2017; Li et al., 2018), which focuses on the visual cortex.

We applied simple and agreed-upon criteria to classify excitatory neurons in the visual cortex (Molnar and Cheung, 2006; Oberlaender et al., 2012). All the identified types based on dendritic morphologies are consistent with previous reports. This could allow our work to be linked to previous studies. With quantitative analysis, we noticed that the apical dendrite height and branches show the natural clustering group (Figure 2C). These are the basis for a clear distinction for different types. Moreover, we have performed both dendritic and axonic-based neuron classification. Correlating the apical dendrite-based neuron types and the projection types, we found partial correlations between dendrites and axon projections. It suggested the significance of incorporating multiple and quantitative features for cell-type classification (Zeng and Sanes, 2017).

Our results were consistent with previous work (Molyneaux et al., 2007; Harris and Shepherd, 2015). We have found that neurons in the visual cortex could project to several areas. The results supported the concept of “one neuron-multiple targets” (Han et al., 2018). The most anterior projection of axons to the orbital area in lateral part, the secondary motor area and anterior cingulate area in dorsal part may account for their secondary activation following visual stimulation or visual cortex stimulation (Lim et al., 2012). These characteristics are related to the possible function of brains. Additionally, the corticothalamic neurons in L6 of visual cortex may have surficial dendrites within L1–L3. The subcerebral projection neurons in L5 of visual cortex (Supplementary Table 1) project to the isocortex, striatum, thalamus, midbrain, medulla, pons, and hypothalamus (Figure 5). The connection between visual area and motor area, anterior cingulate area have been demonstrated by both anterograde and retrograde labeling (Zingg et al., 2014). In our case, an individual L5 neuron in the visual area projects to motor area, anterior cingulate area and multiple subcortical brain regions at the same time (Figure 5A, neuron #92). The single axon-level have presented some unique and striking findings when compared with the previous report (Oh et al., 2014; Zingg et al., 2014). However, there may be some artifact of projection (e.g., axon terminals located in the fiber tracts) resulted from the technical limitation of imaging in current work. No doubt, better labeling and imaging technique will help to decipher the wiring map of brain.

Due to the labeling of the Thy1 H-line of mice, neuron somata are frequently located in layers 5 and 6. The cell type is not as specific but this is an opportunity to study neuronal classification in less mice. An explanation on the small number of animals (*n* = 2) is due to both the nature of conserved anatomy and the consistency of the reported neuron types. Genetic labeling of H-line is not as sparse but can be used for complete reconstruction according to our previous practices. The selection of Thy1-eYFP H-line may benefit us to do comparison with the reported studies in the mouse brain of the same line (Kim et al., 2016; Richter et al., 2018). To compensate the fact that the number of neurons in L5 is smaller than the number in L6 in visual cortex of the Thy1-eYFP H-line transgenic mice, it would be helpful to design experiments from different lines of mice, such as Thy1-eYFP G-line, Thy1-CFP 4-line (Feng et al., 2000) and Sim1_KJ18, Efr3a_NO108, et al. (Gerfen et al., 2013). In the future, more specific genetically labeled neurons may be studied with the same paradigm of this work. How to integrate the morphological, electrical and genetic characterization of neurons will be a critical issue in neuroscience (Gouwens et al., 2018). There is no electrophysiological recording for the neurons in this study. It may be possible to perform cell-type-specific recordings after morphological classification (Economo et al., 2018). We hope that a morphological study could guide further functional studies (Zeng and Sanes, 2017). Additionally, the realistic reconstruction of neurons will be helpful for the modeling and simulation of neural circuits (Markram et al., 2015).

In summary, our high-precision imaging system with single-axon-level reconstruction provides unique and detailed information for long-range projection patterns, which may provide interesting implications for the function of individual neurons.

## Data Availability

The raw morphological data (^∗^.swc files) supporting the conclusions of this manuscript will be made available by the authors, without undue reservation, to any qualified researcher. The data will be shared with community via NeuroMorpho.org once the manuscript is published.

## Ethics Statement

All the procedures followed in the animal experiments were approved by the Institutional Animal Ethics Committee of Huazhong University of Science and Technology. The animal care and use were done in accordance with the guidelines of the Administration Committee of Affairs Concerning Experimental Animals in Hubei Province of China.

## Author Contributions

YZ, SC, and HG designed the study. YZ and SC wrote the manuscript. MR, XL, HW, and JY performed the tissue preparation and whole-brain data acquisition. YZ, ZX, SJ, and AL performed the image processing and visualization. YZ and SJ finished the neuron reconstruction. QL served as project advisor and participated in the planning and organizing of the project.

## Conflict of Interest Statement

The authors declare that the research was conducted in the absence of any commercial or financial relationships that could be construed as a potential conflict of interest.

## Footnotes

1 http://atlas.brain-map.org/
