# Supplementary material for: Pinpointing Morphology and Projection of Excitatory Neurons in Mouse Visual Cortex
Source: Front Neurosci. 2019 Aug 29;13:912. doi: 10.3389/fnins.2019.00912 (PMC6727359; doi:10.3389/fnins.2019.00912)
Supplement: Supplementary file 1 [file Data_Sheet_1.docx]

**Supplementary table and figure legends**

**SUPPLEMENTARY TABLE 1 |** The coordinates of 92 neuronal somata in Allen Mouse Common Coordinate Framework (Allen CCFv3).

| **Neuron number** | **Coordinates of soma in Allen CCFv3 (μm)** | | | **Neuron number** | **Coordinates of soma in Allen CCFv3 (μm)** | | |
| --- | --- | --- | --- | --- | --- | --- | --- |
|  | **x** | **y** | **z** |  | **x** | **y** | **z** |
| 1 | 3107.0 | 2022.9 | 7883.8 | 2 | 3310.4 | 1927.6 | 7860.5 |
| 3 | 3355.7 | 1912.4 | 7803.5 | 4 | 3387.2 | 1860.4 | 7842.0 |
| 5 | 3584.6 | 1759.8 | 7809.0 | 6 | 4101.7 | 1725.8 | 7863.9 |
| 7 | 4119.2 | 1786.4 | 7795.2 | 8 | 2919.1 | 2103.4 | 7976.0 |
| 9 | 2988.0 | 1799.4 | 7910.1 | 10 | 3020.8 | 1820.9 | 7992.2 |
| 11 | 3148.6 | 1992.0 | 7904.4 | 12 | 3233.1 | 1942.0 | 7956.6 |
| 13 | 3804.1 | 1727.9 | 7977.7 | 14 | 2910.1 | 2060.9 | 8111.4 |
| 15 | 2981.3 | 1984.3 | 8064.5 | 16 | 3083.6 | 1945.4 | 8039.1 |
| 17 | 3125.2 | 2007.3 | 8038.3 | 18 | 3183.2 | 1928.2 | 8073.9 |
| 19 | 3116.7 | 1840.2 | 8076.1 | 20 | 3603.1 | 1891.8 | 7988.8 |
| 21 | 3719.6 | 1874.8 | 8095.1 | 22 | 2056.5 | 1983.8 | 8345.5 |
| 23 | 2369.8 | 2165.7 | 8202.6 | 24 | 2669.5 | 2252.5 | 8151.4 |
| 25 | 2925.4 | 1961.2 | 8159.9 | 26 | 3077.4 | 1999.1 | 8139.2 |
| 27 | 4193.4 | 1316.7 | 8166.1 | 28 | 2303.1 | 2479.7 | 8353.7 |
| 29 | 2428.3 | 2369.2 | 8327.8 | 30 | 2520.3 | 2102.8 | 8308.2 |
| 31 | 3113.9 | 1962.1 | 8243.3 | 32 | 3327.4 | 2030.2 | 8274.4 |
| 33 | 3454.1 | 1882.9 | 8299.3 | 34 | 3635.2 | 1895.7 | 8267.1 |
| 35 | 2290.7 | 2311.4 | 8447.5 | 36 | 2509.9 | 2320.1 | 8367.0 |
| 37 | 2464.0 | 2041.5 | 8458.8 | 38 | 3067.5 | 2046.5 | 8334.2 |
| 39 | 3217.5 | 1976.4 | 8340.6 | 40 | 3168.5 | 1714.6 | 8331.6 |
| 41 | 3438.1 | 1874.7 | 8330.2 | 42 | 3568.8 | 1716.7 | 8442.2 |
| 43 | 3623.1 | 1884.4 | 8315.8 | 44 | 3654.1 | 1846.8 | 8308.6 |
| 45 | 3786.6 | 1752.5 | 8407.0 | 46 | 4064.8 | 1590.7 | 8402.0 |
| 47 | 3952.7 | 1275.7 | 8532.4 | 48 | 3933.5 | 1761.0 | 8504.4 |
| 49 | 4002.7 | 1657.9 | 8539.6 | 50 | 2492.8 | 2317.4 | 8927.7 |
| 51 | 2580.3 | 2397.0 | 8865.5 | 52 | 2789.3 | 2119.9 | 8921.6 |
| 53 | 2864.4 | 2228.7 | 8950.0 | 54 | 2872.7 | 2107.9 | 8891.6 |
| 55 | 3093.8 | 2032.2 | 8945.9 | 56 | 3181.1 | 1991.4 | 8955.4 |
| 57 | 3546.0 | 1951.7 | 8950.8 | 58 | 3880.5 | 1692.5 | 8962.3 |
| 59 | 2919.8 | 2198.6 | 9043.9 | 60 | 3064.8 | 2014.4 | 9001.8 |
| 61 | 3172.1 | 2196.2 | 8992.3 | 62 | 3351.8 | 1986.6 | 9065.2 |
| 63 | 3435.3 | 1953.6 | 8974.2 | 64 | 3638.3 | 1617.1 | 9114.8 |
| 65 | 3637.1 | 1866.9 | 9043.7 | 66 | 2975.0 | 2257.7 | 9147.7 |
| 67 | 3028.8 | 2108.5 | 9173.8 | 68 | 3109.6 | 2204.3 | 9122.8 |
| 69 | 3112.5 | 2077.3 | 9242.6 | 70 | 3130.7 | 2126.0 | 9167.4 |
| 71 | 3367.7 | 1947.9 | 9142.1 | 72 | 3368.7 | 2038.5 | 9219.1 |
| 73 | 3683.7 | 1924.4 | 9100.5 | 74 | 3780.2 | 1800.6 | 9193.1 |
| 75 | 3766.5 | 1769.1 | 9198.7 | 76 | 2607.3 | 2523.6 | 9373.7 |
| 77 | 2938.3 | 2259.9 | 9302.8 | 78 | 3205.0 | 1817.6 | 9277.1 |
| 79 | 3353.3 | 2024.7 | 9308.2 | 80 | 3776.7 | 1760.0 | 9329.4 |
| 81 | 2303.1 | 2839.1 | 9493.5 | 82 | 2569.6 | 2594.7 | 9460.2 |
| 83 | 2778.8 | 2385.2 | 9559.4 | 84 | 3400.2 | 2193.3 | 9698.4 |
| 85 | 3493.7 | 2112.6 | 9556.3 | 86 | 2846.1 | 2543.8 | 9889.7 |
| 87 | 3696.2 | 1762.7 | 7814.3 | 88 | 3908.4 | 1795.1 | 7888.6 |
| 89 | 2991.6 | 1718.9 | 7979.4 | 90 | 3393.7 | 1932.1 | 7971.2 |
| 91 | 3603.1 | 1890.7 | 7988.8 | 92 | 3747.0 | 1842.9 | 7997.3 |

**SUPPLEMENTARY FIGURE 1** Neurons of another 8-week-old Thy1-eYFP H-line transgenic male mouse in the visual cortex. **(A)** The 11 neurons with their complete morphologies are shown, and the 6 colors of neurons represent the 6 types defined in **Figure 2**. **(B)** The 11 neurons are shown in the mouse brain with different viewing perspectives.
