# Supplementary figures and images for: Pinpointing Morphology and Projection of Excitatory Neurons in Mouse Visual Cortex
Source: Front Neurosci. 2019 Aug 29;13:912. doi: 10.3389/fnins.2019.00912 (PMC6727359; doi:10.3389/fnins.2019.00912)

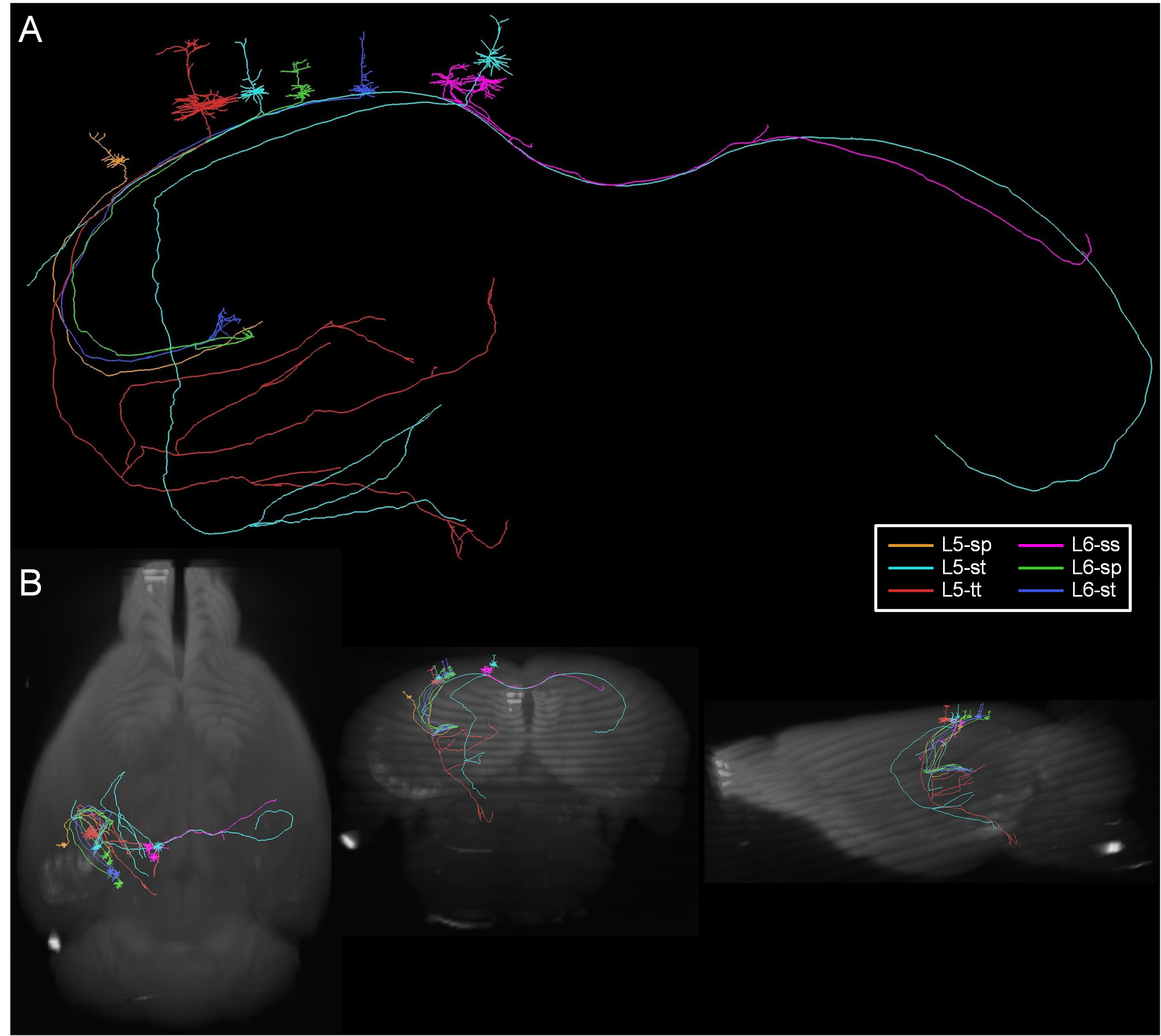

Supplement: Supplementary file 2 [file Image_1.TIF]
